# The interplay between circulating high-density lipoprotein, age and fracture risk: a new cohort study and systematic meta-analysis

**DOI:** 10.1007/s11357-023-00801-w

**Published:** 2023-04-28

**Authors:** Setor K. Kunutsor, Jari A. Laukkanen

**Affiliations:** 1 Diabetes Research Centre, Leicester General Hospital, University of Leicester, Gwendolen Road, Leicester, LE5 4WP UK; 2 Institute of Clinical Medicine, Department of Medicine, University of Eastern Finland, Kuopio, Finland; 3 Institute of Public Health and Clinical Nutrition, University of Eastern Finland, Kuopio, Finland; 4 Department of Medicine, Wellbeing Services County of Central Finland, Jyväskylä, Finland

**Keywords:** High-density lipoprotein cholesterol, Age, Fracture, Cohort study, Systematic review, Meta-analysis

## Abstract

Previous findings on the association between high-density lipoprotein cholesterol (HDL-C) and fracture have yielded inconsistent results and it is unclear if the association varies with age and sex. We sought to evaluate the prospective association between HDL-C levels and fracture risk and assess if the association is modified by age and sex. Circulating HDL-C levels were measured at baseline in a population-based sample of 2,448 men aged 42–61 years. Cox regression was used to estimate hazard ratios (HRs) and 95% CIs. Incident fractures (*n* = 134) occurred during a median follow-up of 25.7 years. In analysis adjusted for several risk factors, the HR (95% CI) for fractures was 1.00 (0.85–1.20) per 1 standard deviation (SD) increase in HDL-C levels**.** Comparing the extreme tertiles of HDL-C levels, the corresponding adjusted HR (95% CI) was 0.94 (0.62–1.45). In a meta-analysis of eight cohort studies (including the current study) comprising 74,378 participants and 4,621 fracture cases, the fully-adjusted risk estimate (95% CI) for fracture was 1.03 (0.96–1.10) per 1SD increase in HDL-C levels and 1.05 (0.92–1.20) comparing extreme tertiles of HDL-C. The pooled risk estimate (95% CIs) for fracture per 1SD increase were 1.09 (1.01–1.17) and 0.98 (0.93–1.04) for age groups ≥ 60 and < 60 years, respectively, and the corresponding risks comparing the extreme tertiles of HDL-C levels were 1.21 (1.09–1.33) and 0.95 (0.85–1.07) (*p*-value for interaction < 0.05). Age may modify the association between HDL-C levels and fracture risk – an increased fracture risk associated with increased HDL-C levels is only evident in older age (≥ 60 years).

**Supplementary Information:**

The online version contains supplementary material available at 10.1007/s11357-023-00801-w.

## Introduction

Fractures (particularly the non-traumatic types) constitute a global public health burden – they are associated with morbidity, disability, poor quality of life, high economic costs and mortality [1]. Though major risk factors such as sociodemographic characteristics, comorbidities, and bone mineral density (BMD) contribute to fracture risk [2], it appears some of the these factors do not explain a large proportion of the risk of fractures. Other factors may contribute to the residual risk of fractures. Unfavourable lipid profiles are major contributors of atherosclerotic cardiovascular diseases (ASCVDs), but they have also been linked to other disease conditions including fractures [3]. High-density lipoprotein cholesterol (HDL-C) is a major lipid. Given preclinical evidence showing that HDL-C reduces bone mineral density [4], it has been hypothesized that high HDL-C levels may be associated with an increased risk of fractures. However, previous evaluations of the associations have yielded inconsistent results. Indeed, some studies have reported positive associations [5], whereas others have reported null results [6]. Given the inconsistent nature of the evidence, there is a need to re-evaluate the evidence. To investigate the detailed nature, magnitude and specificity of the prospective association between HDL-C levels and fracture risk, we (1) utilized a new population-based prospective cohort of men with no previous history of fractures from eastern Finland and (2) conducted a pooled analysis of previous studies (plus the current study) that have evaluated the prospective associations between baseline HDL-C levels and fracture risk. This enabled us to (i) overcome sample size limitations of single cohort studies; (ii) increase precision; and (iii) minimise biases.

## Materials and methods

### Study design and population

The primary cohort study was conducted in accordance with STROBE (STrengthening the Reporting of OBservational studies in Epidemiology) guidelines for reporting observational studies in epidemiology (Electronic Supplementary Material 1). Study participants for the primary cohort analysis were part of the Finnish Kuopio Ischemic Heart Disease (KIHD) population-based prospective cohort study. The study design and recruitment methods have been described in several previous reports [7, 8]. To summarise briefly, a representative sample of 3,433 men aged 42–61 years who were inhabitants of Kuopio city and its surrounding areas in eastern Finland were invited for screening which was carried out between March 1984 and December 1989. Of the 3,433 men, 3,235 were found to be potentially eligible and of this number, 553 did not respond to the invitation or declined to participate and 2,682 men provided consent to participate in the study. For the current analysis, 2448 men had complete information on HDL-C, relevant covariates, and fracture outcomes (Electronic Supplementary Material 2).

### Ethics

The Research Ethics Committee of the University of Kuopio and Kuopio University Hospital, Kuopio, Finland approved the study protocol (License number 143/97). All study procedures were conducted in accordance with the Declaration of Helsinki and written informed consent was obtained from all participants.

### Assessment of HDL-C and relevant risk markers

For measurements of blood biomarkers including lipoproteins, study participants provided blood specimens between 8:00 and 10:00 a.m. after having abstained from alcohol consumption for 3 days, from smoking for 12 h, and after an overnight fast. After a study participant had rested in the supine position for 30 min, blood was drawn with Terumo Venoject VT-100PZ vacuum tubes (Terumo Corp., Tokyo, Japan). No tourniquet was used. The main serum lipoprotein fractions consisting of HDL, low-density lipoprotein (LDL) and very-low-density lipoprotein (VLDL), were separated within three days of blood sampling by a combination of ultracentrifugation and precipitation. The cholesterol content (mmol/L) of all lipoprotein fractions were determined with enzymatic methods (cholesterol CHOD-pap method, Boehringer Mannheim, Mannheim, FRG) [9]. The measurement of pH-corrected serum active calcium concentrations was made using ion selective electrodes (Microlyte 6, Kone, Finland; CV 1.6%) [10]. A random-zero sphygmomanometer was used to measure resting blood pressure; following a supine rest of 5-min, blood pressure was measured three times in supine position, once in a standing position, and twice in a sitting position with 5-min intervals, and the arithmetic mean of all available measurements was taken [11, 12]. Self-administered lifestyle and health questionnaires were used to assess lifestyle characteristics such as smoking, alcohol consumption, physical activity and socioeconomic status (SES), prevalent medical conditions and use of medications [13]. The assessment of SES involved the creation of a summary index based on indicators such as income, education, occupational prestige, material standard of living and housing conditions [14-16]. The composite SES index ranged from 0 to 25, with higher values indicating lower SES. A history of coronary heart disease (CHD) was defined as previous myocardial infarction, angina pectoris, the use of nitroglycerin for chest pain ≥ once a week or chest pain. Energy expenditure of physical activity was assessed using the validated KIHD 12-month leisure-time physical activity questionnaire [17, 18], which was modified from the Minnesota Leisure-Time physical activity Questionnaire [19].

### Ascertainment of incident fractures

The outcome assessed was any non-traumatic fracture (defined as hip, humeral, or wrist fractures) that occurred from study entry to 2014 [8]. Data on incident fractures was collected from the National Hospital Discharge Register data (maintained by the Finnish Institute for Health and Welfare) by computer linkage using Finnish personal identification codes and a comprehensive review of hospital records, discharge diagnoses, and inpatient physician claims. Fracture outcomes were coded according to the International Classification of Diseases Tenth Revision diagnostic codes for fractures by site.

### Statistical analyses

#### Prospective cohort analysis

Baseline characteristics were presented as means (standard deviation, SD) or medians (interquartile range, IQR) for continuous variables and counts (percentages) for categorical variables using descriptive analyses. Hazard ratios (HRs) with 95% confidence intervals (CIs) for fractures were calculated using Cox proportional hazard models after confirmation of no major departure from the proportionality of hazards assumptions using Schoenfeld residuals. High-density lipoprotein cholesterol was modeled as continuous [per 1SD increase] and categorical (tertiles) exposures. Hazard ratios were adjusted for in two models: (model 1) age and (model 2) model 1 plus systolic blood pressure (SBP), history of hypertension, CHD and type 2 diabetes (T2D), smoking status, SES, physical activity, alcohol consumption and circulating calcium. These confounders were selected based on the following criteria: (i) their established roles as risk factors for fractures, (ii) published associations with fracture in the KIHD study [8, 20, 21] or (iii) their potential as confounders based on known associations with fracture outcomes and observed associations with the exposure using the available data [22].

#### Systematic review and meta-analysis

The systematic review protocol was registered in the PROSPERO prospective register of systematic reviews (CRD42023393414) and was conducted in accordance with PRISMA and MOOSE guidelines (Electronic Supplementary Materials 3–4). We searched MEDLINE and Embase from inception to 24 January 2023 for published observational population-based cohort studies that had examined the relation of circulating HDL-C with the risk of fracture events in general adult populations and had at least 1 year of follow-up. Details of the search strategy are reported in Electronic Supplementary Material 5. The risk of bias within individual observational studies was assessed using the Cochrane Risk of Bias in Non-randomised Studies – of Interventions (ROBINS-I) tool [23]. To enable a consistent approach to the meta-analysis and enhance comparison, reported study-specific risk estimates were all transformed to per 1 SD increase and extreme tertiles of HDL-C using standard statistical methods, which have been described previously [7, 8, 24-26]. Summary relative risks (RRs) with 95% confidence intervals (CIs) were pooled using random effects models to minimize the effect of between-study heterogeneity. Fixed effects models were used in parallel analyses. In-between study statistical heterogeneity was quantified using standard chi-square tests and the I^2^ statistic [27]. Given that the risk of fractures varies with age and sex [2], we also assessed if the association between HDL-C and fracture risk is modified by age and sex using stratified analysis and random effects meta-regression [28]. STATA release MP 16 (StataCorp LP, College Station, TX, USA) was used for all statistical analyses.

## Results

Table 1 summarizes the baseline characteristics of the 2,448 participants in the primary cohort study. The mean (SD) of circulating HDL-C was 49.9 (11.7) mg/dl. The follow-up duration ranged from 0.02 to 30.8 years with a median (IQR) follow-up duration of 25.7 (17.5, 27.7) years. During the follow-up period, 134 fractures (annual rate 2.49/1,000 person-years at risk; 95% CI: 2.10–2.94) occurred. In analysis adjusted for age, SBP, history of hypertension, CHD and T2D, smoking status, SES, physical activity, alcohol consumption and circulating calcium, the HR (95% CI) for fractures was 1.00 (0.85–1.20) per 1SD increase in HDL-C levels**.** Alternatively, comparing the top versus bottom tertiles of HDL-C levels, the corresponding adjusted HR (95% CI) was 0.94 (0.62–1.45).

**Table 1:** Baseline characteristics of primary cohort participants (*N* = 2448)

	Mean (SD) or median (IQR)
*Exposure*	
HDL-C (mg/dl)	49.9 (11.7)
*Questionnaire/Prevalent conditions*	
Age at survey (years)	53 (5)
Alcohol consumption (g/week)	32.0 (6.3–93.4)
Socioeconomic status	8.45 (4.24)
History of type 2 diabetes, n (%)	97 (4.0)
Current smokers, n (%)	786 (32.1)
History of CHD, n (%)	620 (25.3)
History of hypertension, n (%)	736 (30.1)
*Physical measurements*	
BMI (kg/m^2^)	26.9 (3.6)
SBP (mmHg)	134 (17)
DBP (mmHg)	89 (10)
Total physical activity (kj/day)	1204 (632–2015)
*Blood-based markers*	
Total cholesterol (mmol/l)	5.90 (1.07)
Fasting plasma glucose (mmol/l)	5.35 (1.25)
Serum ionized calcium (mmol/l)	1.18 (0.05)

*BMI* body mass index; *CHD* coronary heart disease; *CI* confidence interval; *DBP* diastolic blood pressure; *HDL-C* high-density lipoprotein cholesterol; *IQR* interquartile range; *SD* standard deviation; *SBP* systolic blood pressure

**Fig. 1:**
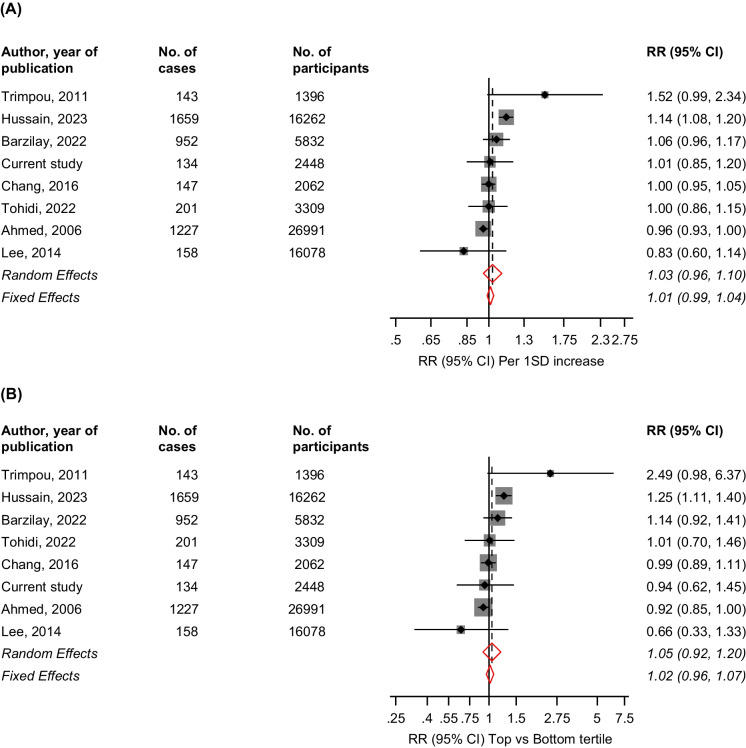
Observational Cohort Studies of HDL-C and Risk of Fractures. (**A**) Per 1 standard deviation increase in HDL-C levels (**B**). Top versus bottom tertiles of HDL-C levels. The summary random and fixed effects estimates presented are based on fully adjusted estimates; CI, confidence interval (bars); HDL-C, high-density lipoprotein cholesterol; RR, relative risk; SD, standard deviation

**Fig. 2:**
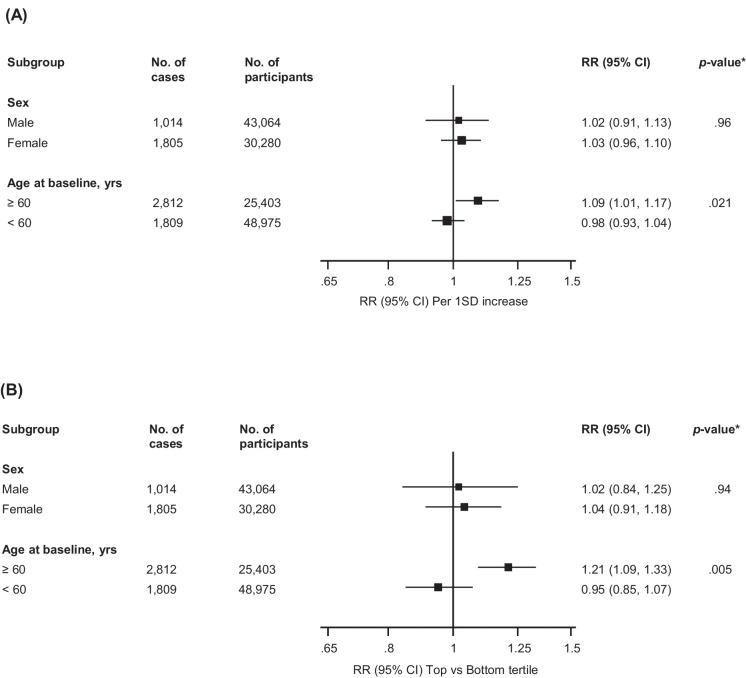
Association Between HDL-C and Fracture Risk According to Age and Sex. (**A**) Per 1 SD increase (**B**) Top vs Bottom tertile of HDL-C. CI, confidence interval; HDL-C, high-density lipoprotein cholesterol; RR, relative risk; SD, standard deviation

In the systematic meta-analysis, we identified seven general population-based prospective cohort studies reporting on the associations between HDL-C levels and incident fracture risk [3, 5, 6, 29-32] (Electronic Supplementary Material 6 and Table 2). Including the current cohort study, the pooled analysis comprised eight studies involving 74,378 participants and 4,621 fracture cases. The average age and HDL-C levels at baseline ranged from approximately 45–75 years and 42.2–61.0 mg/dl, respectively, and the follow-up duration ranged from 2.0–26.0 years. Though there was variation in the degree of covariate adjustment, all eight studies adjusted for established risk factors (Table 2). All eight were at moderate risk of bias using the Cochrane Risk of Bias in Non-randomised Studies – of Interventions (ROBINS-I) tool (Electronic Supplementary Material 7). The pooled multivariable-adjusted random and fixed effects RRs (95% CIs) for fracture per 1SD HDL-C increase were 1.03 (0.96–1.10) and 1.01 (0.99–1.04), respectively; (*I*^*2*^ = 79.2%, 95% CI: 0.0 to 92.8%; *p-value* < 0.001). The corresponding risks comparing the top versus bottom tertiles of HDL-C levels were 1.05 (0.92–1.20) and 1.02 (0.96–1.07), respectively; (*I*^*2*^ = 71.4%, 95% CI: 0.0 to 89.7%; *p-value* = 0.001) (Fig. 1). The pooled random effects RRs (95% CIs) for fracture per 1SD increase were 1.09 (1.01–1.17) and 0.98 (0.93–1.04) for age groups ≥ 60 and < 60 years, respectively, and the corresponding risks comparing the top versus bottom tertiles of HDL-C levels were 1.21 (1.09–1.33) and 0.95 (0.85–1.07), respectively (*p*-value for meta-regression for all < 0.05) (Fig. 2). There was no evidence of effect modification by sex. Using the Grading of Recommendations Assessment, Development and Evaluation (GRADE) tool, the certainty of the evidence was very low (Electronic Supplementary Material 8).

**Table 2:** Baseline characteristics of eligible studies

Author, year of publication	Study name	Country	Baseline year	Mean/median age, yrs	Male, %	Follow-up, yrs	Exposure	Assay	Fasting samples	No. of cases	No. of participants	Fracture outcomes	Adjustment factors
Ahmed, 2006	Tromso	Norway	1994–1995	47.0	47.3	6.0	Serum HDL-C	NR	Non-fasting	1227	26991	Nonvertebral fractures	Age, DM, smoking and physical activity
Trimpou, 2011	Gothenburg WHO MONICA Project	Sweden	1985	44.6	47.0	20.0	Plasma HDL-C	Enzymatic methods	Overnight fast	143	1396	Upper arm, wrist, ankle, leg, hip, pelvis, rib, vertebrae, and foot	Age, sex, time since baseline, BMI and cholesterol
Lee, 2014	South Korean National Claim Registry	Korea	2007–2011	57.1	100.0	3.0	Serum HDL-C	Enzymatic colorimetric	12 h fast	158	16078	Fractures at osteoporosis-related sites	Age, smoking and drinking habits, physical exercise, dairy product consumption, history of stroke, use of drugs that can affect bone metabolism, including bisphosphonates, vitamin D, thyroid hormone, or glucocorticoids, during study periods, and the presence of baseline radiological vertebral fractures
Chang, 2016	SWAN	USA	1995–1997	46.0	0.0	2.0	Plasma HDL-C	Enzymatic methods	12 h fast	147	2062	Foot, ankle, wrist, ribs, and legs	Age, race/ethnicity, study site, menopausal stage, smoking, alcohol use, physical activity, DM, BMI, and lumbar spine BMD
Barzilay, 2022	CHS	USA	1989–1990	73.0	42.5	13.5	Plasma HDL-C	Enzymatic methods	NR	952	5832	Hip	Age, gender, race, smoking, alcohol, HTN, estimated GFR, DM, energy expended per week, frailty, CRP, ADLs, iADLs, weight, height, and prevalent CVD
Tohidi, 2022	TLGS	Iran	1999–2001/2002–2005	60.3	48.3	18.0	Plasma HDL-C	Enzymatic colorimetric method	12–14 h	201	3309	Any fracture	Age, BMI, smoking, T2D, hypertension, and lipid-lowering medication
Hussain, 2023	ASPREE	Australia and USA	2010–2014	75.0	45.0	4.0	Plasma HDL-C	NR	NR	1659	16262	Vertebral, hip or nonhip, nonvertebral	Age, sex, physical activity, alcohol use, prefrailty/frailty status, education, body mass index, smoking status, aspirin use, diabetes, chronic kidney disease, use of lipid-lowering medication, and use of antiosteoporosis medications
Current study	KIHD	Finland	1984–1989	53.0	100.0	25.7	Plasma HDL-C	Enzymatic methods	Overnight fast	134	2448	Hip, humeral, or wrist fractures	Age, SBP, history of hypertension, history of CHD, smoking, history of DM, SES, physical activity, alcohol consumption and circulating calcium

*ADL* activities of daily living; *BMD* bone mineral density; *BMI* body mass index; *CHD* coronary heart disease; *CRP* C-reactive protein; *CVD* cardiovascular disease; *DM* diabetes mellitus; *GFR* glomerular filtration rate; *HDL-C* high-density lipoprotein cholesterol; *HTN* hypertension; *NR* not reported; *SBP* systolic blood pressure; *SES* socioeconomic status; *T2D* type 2 diabetes

Study abbreviations: *ASPREE* Aspirin in Reducing Events in the Elderly’; *CHS* Cardiovascular Health Study; *KIHD* Kuopio Ischemic Heart Disease; *SWAN* Study of Women’s Health Across the Nation; *TLGS* Tehran Lipid and Glucose Study

## Discussion

Our primary analysis of a population-based prospective study of middle-aged and older Finnish men demonstrated no evidence of an association between HDL-C levels and fracture risk. Pooled analysis of seven previous studies plus the current study using random or fixed effects models showed no evidence of an association. However, stratified analysis by age showed significant evidence of an increased risk of fracture with increased HDL-C levels in participants ≥ 60 years, with no evidence of an association in the younger age group (< 60 years).

In the most recent evaluation of the relationship between HDL-C and fracture risk, Hussain and colleagues conducted a posthoc analysis of the Aspirin in Reducing Events in the Elderly (ASPREE) trial comprising 16,262 participants aged ≥ 65 years and their results suggested that HDL-C might be an independent risk marker for fracture risk [5]. Their results showed a linear relationship between HDL-C and risk of fractures for both male and female participants [5]. The current findings add to the controversy about the nature of the epidemiological associations between HDL-C and outcomes. High-density lipoprotein cholesterol is considered an established risk factor for ASCVD and has a consistent, independent, strong, graded and inverse relationship with adverse cardiovascular outcomes [33]. High-density lipoprotein cholesterol has always been regarded as the “good cholesterol” with high levels conferring a protective effect and vice versa. However, emerging evidence suggest that higher levels of HDL-C are also associated with increased risk of adverse cardiovascular outcomes, with the association being consistent with a U-shaped relationship [34]. Similar relationships have been demonstrated for other outcomes such as age-related macular degeneration [35]. Barzilay and colleagues in their evaluation of the associations of conventional lipid and lipoprotein levels with incident fracture risk, demonstrated a non-linear U-shaped relationship between HDL-C and hip fracture risk [6]. There have been efforts to explain these conflicting results, but these have mainly been speculative. This is complicated by the fact that HDL and its functional biology and metabolism are complex. Hussain and colleagues suggested the most likely pathophysiological explanation for their findings could be the link between HDL-C levels and BMD [5]; HDL-C has been reported in preclinical studies to reduce BMD via reduction in osteoblast production [36]. A recent study has also suggested that HDL-C might be causally related to fracture risk [37]. The lack of significant evidence of an association between HDL-C levels and fracture risk in the primary cohort could be due to the fact the cohort was based on male participants, who have a lower predisposition to osteoporotic fractures than women [38]. Furthermore, this may be due to inadequate statistical power given the low event rate. Conversely, the null association demonstrated in the primary cohort may be a true one, reflecting the fact that increased levels of HDL-C might only increase the risk of fractures in older people. The mechanisms by which increased HDL-C levels might increase fracture risk in older age are unclear; however, it may be related to the fact that older age is a major risk factor for increased risk of fractures. Furthermore, aging is also known to alter HDL composition resulting in structural and functional impairment [39]; for example, it has been shown that HDL isolated from older people have a reduced capacity to inhibit LDL oxidation and promote cholesterol efflux from macrophages [40, 41]. It has been reported that the structural and functional changes in HDL in older people may be linked to a loss in its potential anti-atherogenic properties, contributing to the high burden of ASCVD in older people [39].

Based on a pooled analysis of all studies conducted on the topic, our findings do suggest that HDL-C may potentially be used as a biomarker to estimate the increased risk of fractures in older participants. It is known that cardiovascular risk estimation varies with different HDL levels [42] and HDL-C levels are also known to decrease with aging [43], hence, the prognostic ability of HDL-C for CVD is expected to change with age. This goes to suggest that the potential prognostic relevance of HDL-C for fracture risk may also vary with age. Nevertheless, these findings and their potential implications are still early and need to be considered in light of the fact that HDL is a complex molecule and its epidemiology may vary with age, gender, alcohol consumption, metabolic status, presence and type of comorbidities, ethnicity, follow-up durations and endpoints. With regards to the current findings, further research is needed to clarify whether the associations of HDL particles and its functional measures with fracture risk vary with age.

## Strengths and limitations

Strengths of this evaluation included the use of a new prospective study and systematic meta-analysis of all available studies to re-evaluate the relationship, enhanced power and the ability to harmonise the data into consistent comparisons to enhance pooling. Most of the limitations were inherent and included the substantial variation in study designs and population and the potential for biases such as residual confounding, reverse causation bias and regression dilution.

## Conclusions

New evidence based on a systematic meta-analysis of available observational prospective cohort studies plus a new population-based cohort study suggest age may modify the association between HDL-C levels and fracture risk – an increased risk of fracture associated with increased HDL-C levels is only evident in older groups (≥ 60 years).

### Supplementary Information

Below is the link to the electronic supplementary material.

Supplementary file1 (DOCX 200 KB)

## Data Availability

The data that support the findings of this study are available from the corresponding author upon reasonable request.
